# The potential protective role of carotenoids from saffron: A focus on endoplasmic reticulum stress‐related organ damage

**DOI:** 10.1002/fsn3.4289

**Published:** 2024-06-21

**Authors:** Farshad Mirzavi, Arezoo Rajabian, Hossein Hosseini

**Affiliations:** ^1^ Cardiovascular Diseases Research Center Birjand University of Medical Sciences Birjand Iran; ^2^ Neuroscience Research Center Mashhad University of Medical Sciences Mashhad Iran; ^3^ Department of Neuroscience Faculty of Medicine, Mashhad University of Medical Sciences Mashhad Iran; ^4^ Department of Clinical Biochemistry Faculty of Medicine, Mashhad University of Medical Sciences Mashhad Iran

**Keywords:** apoptosis, endoplasmic reticulum stress, oxidative stress, saffron carotenoids

## Abstract

The anticancer, antioxidant, and immunomodulatory properties of carotenoids from saffron or apocarotenoids (e.g., crocin, safranal, crocetin, and picrocrocin) have prompted research into their benefits. Apocarotenoids seem to be effective compound for the treatment of chronic diseases, such as neurodegenerative, cardiovascular, cancer, respiratory, and metabolic disorders. Endoplasmic reticulum (ER) is an essential organelle found in the cytoplasm of eukaryotic cells that participates in the biosynthesis of proteins, lipids, and steroid hormones. Given the role of the ER in the regulation of several fundamental biological processes, including metabolic pathways and immune responses, aberrant ER function can have a significant influence on these vital processes and result in serious pathological consequences. Exposure of cell to adverse environmental challenges, such as toxic agents, ischemia, and so on, causes accumulation of unfolded or misfolded proteins in the ER lumen, also called ER stress. There is a growing evidence to suggest that ER disturbance in the form of oxidative/nitrosative stress and subsequent apoptotic cell death plays major roles in the pathogenesis of many human diseases, including cardiovascular diseases, diabetes mellitus, neurodegenerative diseases, and liver diseases. Apocarotenoids with their unique properties can modulate ER stress through PERK/eIF2α/ATF4/CHOP (protein kinase R (PKR)‐like ER kinase/eukaryotic initiation factor 2α/activating transcription factor 4/C/EBP /homologous protein) and X‐Box Binding Protein 1/activating transcription factor 6 (XBP1/ATF6) pathways. In addition, they suppress apoptosis through inhibition of endoplasmic and mitochondrial‐dependent caspase cascade and can stimulate SIRT1 (silent information regulator 1) and Nrf2 (nuclear factor erythroid 2‐related factor 2) expression, thereby leading to protection against oxidative stress. This review summarizes the potential benefits of apocarotenoids in various ER‐stress‐related disorders.

## Graphical abstract

In this review, the experimental evidence on apocarotenoids targeted endoplasmic reticulum (ER) in different models of disease as well as outline how these phytochemicals regulate ER function in the pathological conditions are critically discussed. This helps in designing novel therapeutics for such disorders. We specifically focused on neurological and cardiovascular diseases, cancers, metabolic, ocular, bone, and respiratory disorder as well as toxic‐induced organ damage, which are closely associated with oxidative damage and subsequent apoptotic cell death.

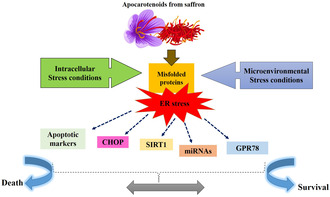

## 1 INTRODUCTION

*Crocus sativus* L., also known as saffron, is a member of the Iridaceae family, which is a perennial flowering plant (Leone et al., 2018). The dried stigma of the flowers has been used in traditional medicine as a curative compound and as a food additive and coloring agent (Cardone et al., 2020). Apocarotenoids, including crocetin, crocin, picrocrocin, and safranal, are found in high content in the stigma of saffron (Avila‐Sosa et al., 2022). Based on the extraction method, these biologically active compounds exhibited a yield of 52%–85% of the saffron compounds (Avila‐Sosa et al., 2022; Leone et al., 2018).

**FIGURE 1:**
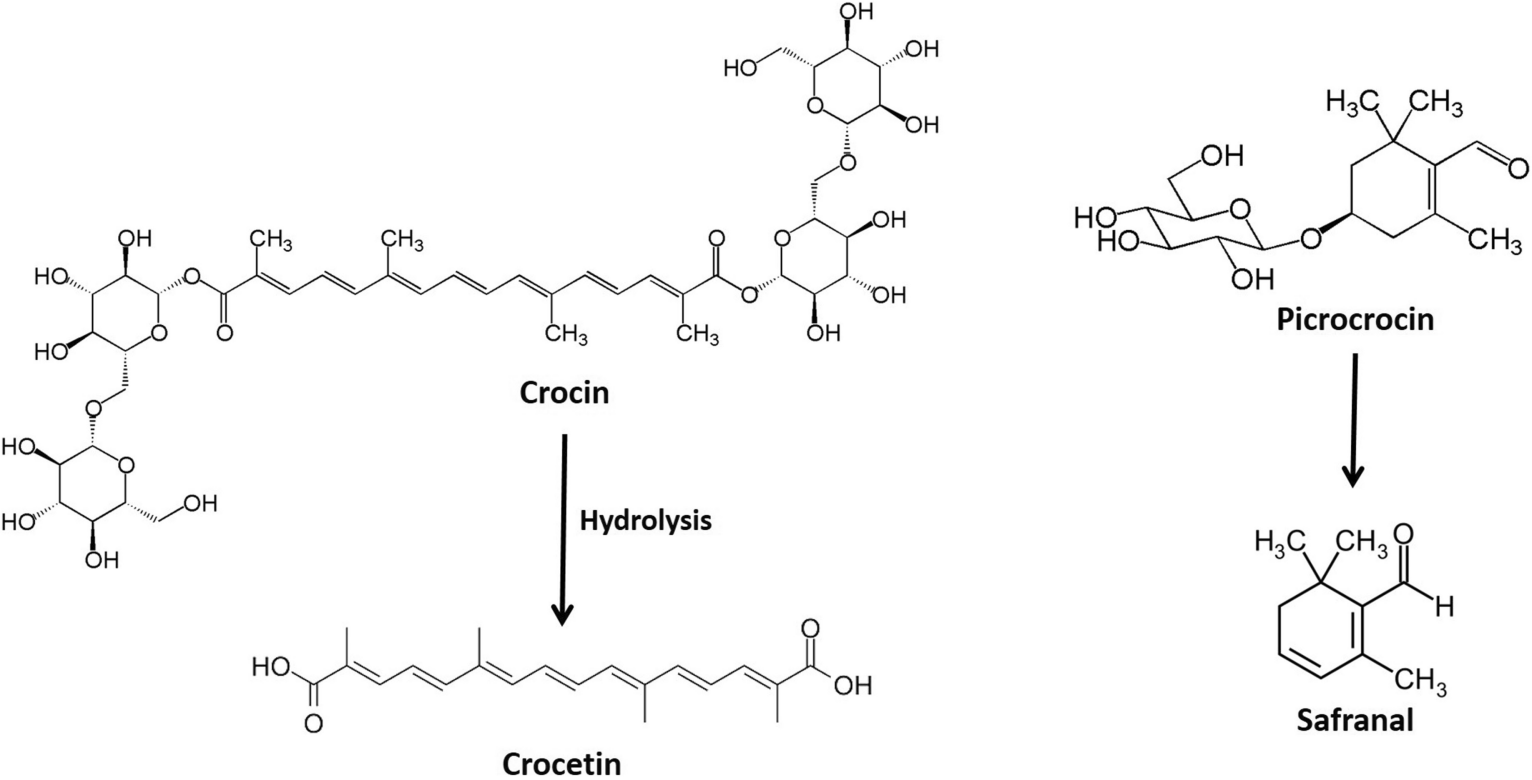
The illustration of the chemical structures of apocarotenoids (crocin, safranal, crocetin, and picrocrocin).

Over the past half a century, apocarotenoids have been shown a growing interest in a wide range of pharmacological investigations (Li et al., 2023). A large number of clinical and preclinical researches have suggested the potential therapeutic properties of these phytochemicals in different pathological conditions (Ghaffari & Roshanravan, 2019; Leone et al., 2018) due to their antioxidant, antitumor (Su et al., 2023), lipid‐modifying (Correia et al., 2023), hepatoprotective, vasculoprotective, cardioprotective, pulmonoprotective, neuroprotective, antithrombotic, immunomodulatory, antidiabetic, analgesic, and anti‐inflammatory properties (Ghaffari & Roshanravan, 2019; Leone et al., 2018). Mechanistically, the interaction of apocarotenoids with various nuclear factors, transcription factors (Imtiaz et al., 2023), hormones, growth factors, and their receptors underlie the variety of their pharmacological activities (Ghaffari & Roshanravan, 2019; Mykhailenko et al., 2019). Among *C. sativus* L. compounds, crocin and crocetin have low solubility and poor absorption. After oral administration, crocin is rapidly hydrolyzed to crocetin in the intestinal epithelium and can be rapidly absorbed across the intestinal barrier (Belyagoubi‐Benhammou et al., 2023). Figure 1 presents an illustration of the chemical structures of apocarotenoids (crocin, safranal, crocetin, and picrocrocin).

Endoplasmic reticulum (ER) is an important organelle in the cytoplasm of eukaryotic cells (Wu et al., 2023). This dynamic organelle consists of rough and smooth ER domains. ER participates in the biosynthesis of proteins as well as lipid and steroid hormone. It also acts as an intracellular calcium (Ca^2+^) reservoir playing a key role in cytosolic Ca^2+^ hemostasis (Ghemrawi & Khair, 2020). Given the role of the ER in the regulation of several biological processes, aberrant ER function can lead to serious pathological consequences, such as neurodegenerative, cardiovascular, cancer, metabolic disorders, inflammatory disorders, and allergic asthma (Ghemrawi & Khair, 2020; Jeong et al., 2017). Hence, ER function may be employed as a biomarker for the prognosis and diagnosis of diseases and as an effective therapeutic target (Yu et al., 2021). Previous evidence from cellular and animal experiments has revealed that various pharmacological effects of apocarotenoids on brain, ocular, renal, heart, pancreas, and liver diseases could be mediated through modifying ER function (Leone et al., 2018; Razak et al., 2017). Apocarotenoids with their unique properties can modulate ER stress through various signaling pathways. Apocarotenoids have been shown to suppress apoptosis through inhibition of the endoplasmic and mitochondrial‐dependent caspase cascade and can stimulate the expression of SIRT1 (silent information regulator 1) and Nrf2 (nuclear factor erythroid 2‐related factor 2), thereby leading to protection against oxidative stress (Wang et al., 2019). The aim of this review is to summarize experimental evidence on apocarotenoids targeted ER in different models of disease as well as outline how these phytochemicals regulate ER function in the pathological conditions. In this review, databases, including PubMed, Web of Science, Scopus, and Google Scholar, were electronically searched for English‐published articles, using the following keywords: “endoplasmic reticulum stress,” “saffron carotenoids,” “crocin,” “crocetin,” “safranal,” “anti‐inflammatory,” “antioxidant,” “oxidative stress,” and “apoptosis.” Related articles published between 2004 and 2023 were reviewed according to the inclusion criteria. This helps in designing novel therapeutics for such disorders. We specifically focused on neurological and cardiovascular diseases, cancers, metabolic, ocular, bone, and respiratory disorder as well as toxic‐induced organ damage, which are closely associated with oxidative damage and subsequent apoptotic cell death.

## 2 ER‐STRESS‐RELATED MARKERS

Evidences showed that many pathological changes are linked to impaired function of ER. Hence, this organelle is involved in normal physiological functions as well as pathophysiological changes including tumors, ischemia, neurodegenerative and metabolic diseases (Ghemrawi & Khair, 2020; Wang et al., 2020). The chaperones and enzymes involved in synthesis and folding of proteins are highly sensitive to the stress conditions, such as disturbance in cellular Ca^2+^ concentration, oxidative stress, and cellular energy depletion. As a result, when cells are exposed to harmful environmental stressors, such as ischemia, viral infection, toxics, pH fluctuations, etc., misfolded/unfolded proteins accumulate in the ER, known as ER stress (Ghemrawi & Khair, 2020; Wang et al., 2020). A protective mechanism, named as unfolded protein response (UPR), modulates ER function to restore ER homeostasis through specific intracellular signaling pathways (Ghemrawi & Khair, 2020). Under stress condition, glucose‐regulated protein 78 kDa/binding immunoglobulin protein (Grp78/BiP) induces UPR through activating three ER transmembrane proteins, such as activating transcription factor 6 (ATF6), inositol‐requiring enzyme 1α (IRE‐1α), and protein kinase R (PKR)‐like ER kinase (PERK). Hence, Grp78 (BiP) is considered as a central regulator of the UPR‐mediated degradation of unfolded proteins (Ghemrawi & Khair, 2020; Wang et al., 2020). Activation of the UPR sensors promotes X‐Box Binding Protein 1 (XBP1), activating transcription factor 4 (AFT4) and AFT6 functions. As the main transcription factors involved in ER secretory pathway, they reduce protein folding, enhance antioxidant responses and gene expression related to cell survival (e.g., autophagy), and promote ER‐associated degradation (ERAD) (Ghemrawi & Khair, 2020; Yarmohammadi et al., 2021).

Activation of PERK/eukaryotic initiation factors (eIFs) 2 phosphorylation (P)/ATF4 pathway activates ATF6, leading to elevation of genes involved in apoptosis, including the pro‐apoptotic factor growth arrest and DNA damage‐inducible gene 153 (GADD153) or CHOP (C/EBP homologous protein) (Ghemrawi & Khair, 2020). Postmortem brain tissues of patients with neurodegenerative diseases were found to contain multiple UPR markers, such as phospho‐PERK (p‐PERK), activated ATF6, phospho‐inositol‐requiring enzyme 1 (p‐IRE1), phospho‐eukaryotic initiation factor 2 (p‐eIF2), binding immunoglobulin protein (BiP), and CHOP, which were associated with the accumulation of aggregated and misfolded proteins (Yarmohammadi et al., 2021). PERK‐mediated apoptosis is a consequence of UPR failure due to strong and sustained causative stimuli of ER stress. Apoptosis is induced by the essential downstream proteins that include CHOP, c‐Jun N‐terminal Kinase (JNK), and ER stress‐specific caspase‐12. As mentioned earlier, CHOP, which is typically generated by genotoxic stress and growth arrest signals, is one of the components of the ER stress‐mediated apoptosis pathway (Yarmohammadi et al., 2021). ER stress may also induce autophagy through the PERK/eIF2α/ATF4 (protein kinase R (PKR)‐like ER kinase/eukaryotic initiation factor 2α/activating transcription factor 4) signaling pathway. Autophagy‐related gene 5 (ATG5) and autophagy‐related gene 7 (ATG7) were shown to link autophagy with ER stress through PERK signaling. ATF4 has been shown to direct the induction of autophagy gene transcription, such as microtubule‐associated protein 1 light chain 3 beta (MAP1LC3β), Beclin‐1 (BECN1), ATG5, ATG7, and autophagy‐related gene 12 (ATG12) in response to ER stress (Ghemrawi & Khair, 2020). A large body of evidence showed that ER stress and UPR can disturb immunity and inflammatory responses (Grootjans et al., 2016). During ER stress, pathological changes in mitochondria (such as free radicals' overload, mitochondrial DNA injury, adenosine triphosphate (ATP) depletion, and Ca^2+^ disturbance) can activate cytosolic NLRP3 (nucleotide‐binding domain, leucine‐rich‐containing family, pyrin domain‐containing‐3) inflammasome. The interaction between mitochondria, ER stress, and the NLRP3 inflammasome may play an important role in the pathogenesis of severe corticosteroid‐resistant type 2 immune response (Ghemrawi & Khair, 2020; Jeong et al., 2017).

## 3 APOCAROTENOIDS COUNTERACT ER DYSFUNCTION IN NEURODEGENERATIVE DISORDERS

Apocarotenoids have a low molecular weight, making them capable of penetrating the blood–brain barrier (Lautenschläger et al., 2015). Increasing evidence have suggested the neuroprotective potential of apocarotenoids in brain injury during ischemic stroke, neurodegenerative disorders, and chemicals‐induced toxicity that were linked to their anti‐inflammatory and antioxidant activities (Leone et al., 2018; Rajabian et al., 2019). Persistent ER stress within central nervous system (CNS) has been suggested as the common pathological characteristic of many neurodegenerative diseases such as multiple sclerosis (MS) (Sprenkle et al., 2017). Prolonged ER stress is believed to disrupt UPR, leading to the activation of apoptotic and inflammatory pathways (Ghemrawi & Khair, 2020). MS is a neurodegenerative disease characterized by demyelination, axonal degeneration accompanied by oligodendrocyte and neuronal death (Ruiz et al., 2019). During MS, reactive peripheral humoral and innate immune cells create a neurotoxic microenvironment through releasing a variety of inflammatory mediators, including reactive oxygen species (ROS), nitric oxide (NO), and inflammatory cytokines, as well as activating astrocytes and microglia (Ruiz et al., 2019; Sprenkle et al., 2017). Along with PERK, IRE‐1α through adaptor protein tumor‐necrosis factor‐α (TNF‐α)‐receptor‐associated factor 2 (TRAF2) activates different inflammatory pathways such as mitogen‐activated protein kinase (MAPK) and nuclear factor kappa B (NF‐κB) signaling pathways (Ghemrawi & Khair, 2020; Jeong et al., 2017). Activation of MAPK and NF‐κB signaling pathways plays a central role in the initiation of innate immune response during UPR (Jeong et al., 2017). NF‐κB regulates different components of inflammatory events such cyclooxygenase‐2 (COX‐2), TNF‐α, caspase family of proteases, and inducible nitric oxide synthase (iNOS), which consequently results in neuron death (Laurindo et al., 2023). Upregulated UPR markers, such as GRP78 and CHOP in MS lesions, support this idea that failure of the UPR‐protective mechanisms triggers neurotoxicity through exacerbating inflammatory responses and apoptotic pathways (Antony et al., 2011). During ER stress, IRE‐1α can facilitate apoptosis through activation of caspase‐12 (Ghemrawi & Khair, 2020). In an experimental setting of autoimmune encephalomyelitis (EAE), crocin counteracted ER stress and inflammation in C57BL/6 wild and CHOP 2/2 mice. As evident from the biochemical assessments, gene expression of XBP‐1 spliced variant (XBP‐1/s), BiP, PERK, and CHOP, and TNF‐α was remarkably suppressed in the spinal cords of EAE mice treated with crocin (100 mg/kg). Further in vitro studies revealed that treatment of human fetal astrocytes with 200 mM of crocin attenuated Syncytin‐1. These findings demonstrate that crocin induces neuroprotection through modulating gene expression of ER stress‐related sensors. Furthermore, crocin can scavenge free radicals and provide the antioxidant state (Yaribeygi et al., 2018) that involved in its protective effects against ER disruption, and subsequent demyelination and neurodegeneration in MS (Deslauriers et al., 2011). In an AD rat model induced by amyloid β (Aβ)_25–35_, crocin (40 mg/kg) exhibited ameliorative effects on learning and memory acquisition using Y‐maze test. It has been shown that crocin counteracts ER stress and subsequent apoptosis of hippocampal and cortical neurons through regulating the expression of GRP78 and CHOP (Lin et al., 2019).

**FIGURE 2:**
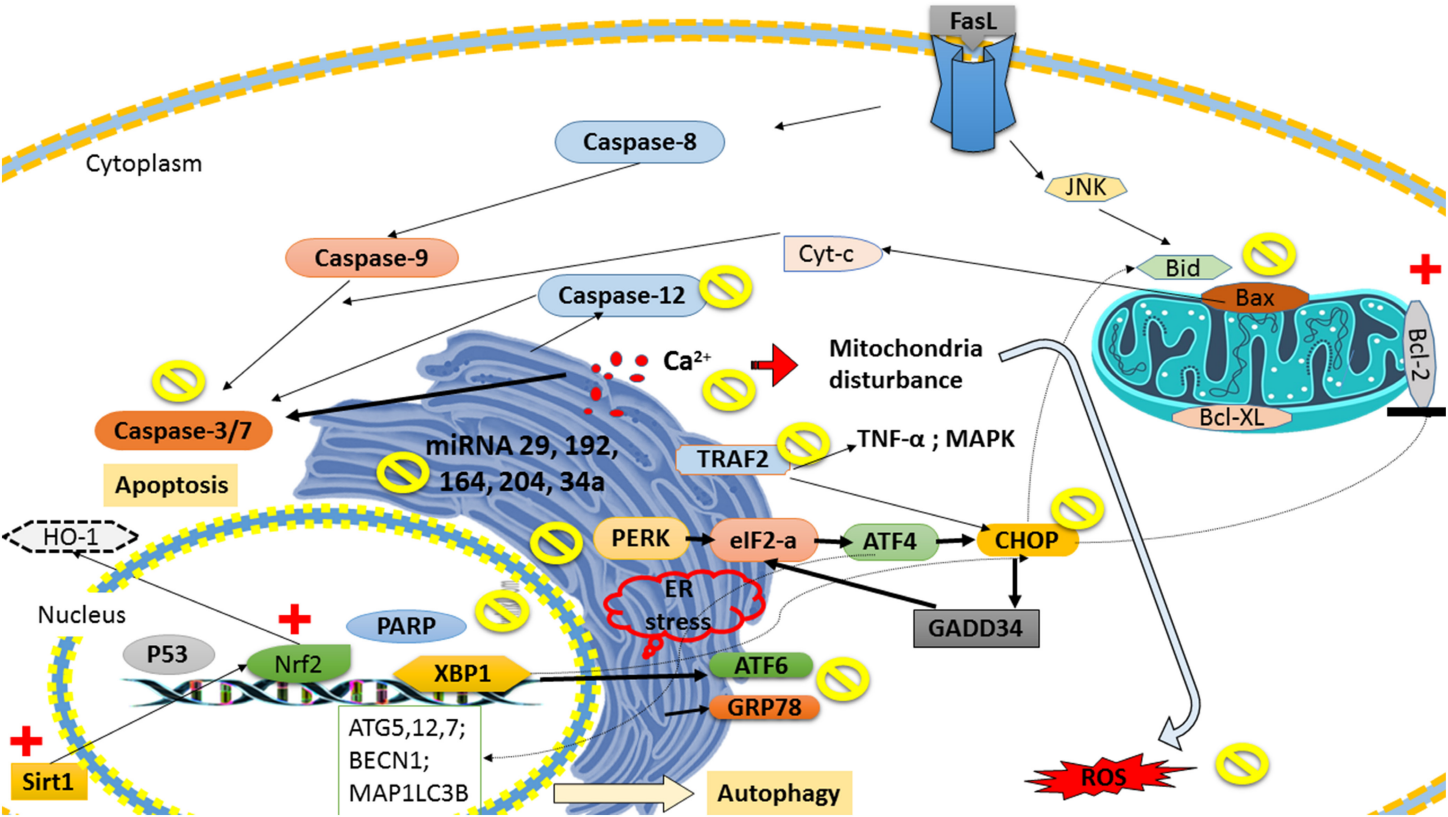
The effect of apocarotenoids on ER stress‐related molecular pathways. Apocarotenoids modulate endoplasmic reticulum stress through PERK/eIF2α/ATF4/CHOP and XBP1/ATF6 pathways. These phytochemicals suppress apoptosis through inhibiting endoplasmic and mitochondrial‐dependent caspase cascade. They also stimulate SIRT1 and Nrf2 expression that consequently leads to protection against oxidative stress. The protective properties of apocarotenoids are also linked to modulation of miR‐204, miR‐216, miR‐192, miR‐29, and miR‐34a expression.

The ameliorative effects of apocarotenoids against neurodegeneration have also been highlighted by other researchers (Bie et al., 2011; Lakey‐Beitia et al., 2019; Yuan et al., 2023). Using oxygen glucose‐deprived SH‐SY5Y cells, as a cerebral ischemia/reperfusion (I/R)‐like condition as well as a rat model of vascular dementia, Yuan et al. (2023) found that crocetin notably mitigated neuronal injury. Suppression of the PERK/eIF2α/ATF4/CHOP pathway was proposed as the probable mechanism involved in the neuroprotective potential of crocetin against neuronal apoptosis during dementia (Yuan et al., 2023). In rat with traumatic brain injury, Bie et al. (2011) proposed that crocetin (500 mg/kg, oral) provides great benefits against neuronal apoptotic cell death that was associated with upregulation of B‐cell lymphoma 2 (Bcl‐2) (Bie et al., 2011). Bcl‐2 was suggested to induce its antiapoptotic effects through suppression of several factors promoting the release of the Ca^2+^ from ER (Bie et al., 2011; Vervliet et al., 2016). Another previous study showed that crocin (0.1, 1, 10, or 100 μM) exerts its protective effects against 1‐methyl‐4‐phenylpyridinium (MPP^+^, 500 μM)‐induced injury partially through regulating CHOP/Wnt (wingless‐related integration site) pathway in the neuron‐like PC12 cells. Interestingly, the function of pro‐survival Wnt/β‐catenin pathway is inversely linked to CHOP expression (Zhang et al., 2015). Hence, crocin is suggested to ameliorate ER‐related apoptosis and consequently increase the neuronal cell survival (Zhang et al., 2015). Considering antioxidant properties of apocarotenoids, some scholars investigated whether the administration of apocaroteniods can mitigate neurotoxicity induced by toxic elements and drugs (Ibrahim et al., 2021). In this regard, saffron aqueous extract was suggested to attenuate side effects of Sofosbuvir, an anti‐hepatitis C drug (Ibrahim et al., 2021). The findings obtained from transmission electron microscopy (TEM) of Purkinje neuron revealed that the saffron extract administration (80 mg/kg /day, for 6 weeks) improved pathological changes in rough ER. The antioxidant and detoxifying capacities of the saffron extract were suggested to be involved in the protective effects against Sofosbuvir‐induced neurotoxicity by attenuating oxidative stress (41.1 mg/kg/day for 6 weeks) (Ibrahim et al., 2021). Increase in lipid peroxidation has been identified as an early and sensitive consequence of copper oxide nanoparticles' toxicity (Anreddy, 2018). Increased lipid peroxidation causes the depletion of antioxidant defense system including glutathione peroxidase (GPx), catalase, superoxide dismutase (SOD), and nonenzymatic molecules, namely glutathione, which normally maintain biological systems from contingent free radical toxicity (Ahmed et al., 2008; Bhagat & Ingole, 2016). The results of a study conducted by Mohamed Mowafy et al. (2021) found that crocin (30 mg/kg/day for 14 days) can effectively inhibit lipid peroxidation and improve antioxidant capacity in brain of rats exposed to copper oxide nanoparticles. They found that the antioxidant effects of crocin ameliorate Purkinje cells of cerebellar cortex histopathological and ultrastructural alterations (including ribosomes, mitochondria, and rough ER) (Mohamed Mowafy et al., 2021). Overall, apocarotenoids showed potential protective effects in MS through modulating XBP‐1 spliced variant (XBP‐1/s), BiP, PERK, CHOP, and Syncytin‐1 in spinal cord. They also modulated GRP78 and CHOP in cerebral tissue of AD mouse associated with amelioration of cognition. Furthermore, regulation of the PERK/eIF2α/ATF4/CHOP pathway was proposed as protective mechanism of apocarotenoids against brain ischemic injury. These phytochemicals were found to prevent cell death during neurodegeneration through regulating CHOP/Wnt pathway. Upregulation of Bcl‐2 is also another protective mechanism of apocarotenoids against neuronal apoptotic cell death. Apocarotenoids are also found to attenuate oxidative injury due to their free radical scavenging and upregulating antioxidant enzymes. Hence, oxidative stress‐provoked apoptotic markers may be modulated by these phytochemicals. Figure 2 illustrates the effect of apocarotenoids on ER stress‐related molecular pathways.

### 3.1 Apocarotenoids counteract ER dysfunction in diabetes

During diabetes, the persistent hyperglycemia is accompanied by both the macro‐ and microvascular complications leading to multiple organ failure (Marcovecchio et al., 2011; Paul et al., 2020). In general, diabetes disrupts several pivotal cellular events involved in the development of diabetic complications, such as redox hemostasis, inflammatory mediators, ER and mitochondria function, which are associated with the activation of transcription factors and enzymes (e.g., protein kinase‐c (PKC)), and advanced glycation end products (AGEs) accumulation in some tissues (Paul et al., 2020). In streptozotocin (STZ)‐induced diabetic rats, aqueous extract of saffron exerted hypoglycemic and hepatoprotective effects. This has been manifested to occur through suppression of blood glucose and normalization of the liver and serum total lipids, triglyceride (TG), cholesterol (TC), low‐density lipoprotein (LDL), and very‐low‐density lipoprotein (VLDL), aspartate and alanine aminotransferase, and total oxidative status (malondialdehyde (MDA) and hydrogen peroxide (H_2_O_2_) level), dysregulated following administration of STZ (50 mg/kg, i.p.). According to ultrastructural observations using electron microscopy, supplementation with the extract ameliorated the structural integrity of the intracellular organelles including mithochondria and ER in hepatocytes of the diabetic rats (Ali et al., 2016). Generally, nuclear factor erythroid‐2‐related factor 2 (Nrf2)/antioxidant response element pathway is considered as a compensatory mechanism activated in the cells exposed to oxidative stress (Moratilla‐Rivera et al., 2023). Nrf2 maintains the cell homeostasis and reinforces the antioxidant defense system. Considering β‐cells have high sensitivity to oxidative damage, the role of Nrf2 signaling pathway has been highlighted in the management of diabetes (Hashemi et al., 2023). Therefore, activation of Nrf2 signaling pathway can reduce the risk of developing diabetes through attenuating the level of oxidative stress (Shaw & Chattopadhyay, 2020). Considering the key role of ER function in the synthesis and secretion of insulin, ER dysfunction develops diabetes (Stone et al., 2021). MicroRNAs (miRs or miRNAs) importantly affect the β‐cell function and regulate response to insulin. MiRNAs, also known as short‐noncoding RNAs (21–25 nucleotides in length), inhibit gene expression of specific messenger RNAs (mRNAs). Hence, they posttranscriptionally regulate various cellular functions including proliferation, differentiation, and apoptosis. They also modulate the ER stress (Ismail et al., 2023; Pathomthongtaweechai & Chutipongtanate, 2020). During hyperglycemia, miR‐29a is increased in the β‐cells and prevents the progression of diabetes (Chen et al., 2014; Pathomthongtaweechai & Chutipongtanate, 2020). miR‐192 is also increased in a prediabetes state and modulates proliferation and insulin secretion. Another miRNA, named miR‐204, is regarded as a specific biomarker of β‐cells' degeneration and can directly target PERK, as a sensor of ER stress. Similarly, miR‐216b was found to be upregulated during pancreatic injury (Feng et al., 2016; Pathomthongtaweechai & Chutipongtanate, 2020). A previous study demonstrated that administration of crocin (15, 30, and 60 mg/kg) to methylglyoxal‐induced diabetic mice ameliorated β‐cell function as indicated by ameliorating fasting blood glucose and redox hemostasis including MDA, glutathione levels, and glyoxalase 1–Nrf2. Meanwhile, crocin decreased miR‐204, miR‐216b, miR‐192, and miR‐29a expression suggesting its inhibitory effects on the progression of diabetes through modulating ER stress (Radmehr et al., 2022). The results of this study further support the idea that miRNAs can affect Nrf2, thereby increasing the protective capacity of the cells against methylglyoxal‐induced oxidative injury (Radmehr et al., 2022).

## 4 THE PROTECTIVE POTENTIAL OF APOCAROTENOIDS IN ER STRESS‐RELATED CARDIOVASCULAR DISORDERS

Crocin has been shown to protect against I/R‐induced heart injury through targeting Sirtuin1 (silent information regulator 1 SIRT1) and miR‐34a in both primary cardiomyocytes and mouse models of I/R (Wang et al., 2019). SIRT1 is a member of the sirtuin family of nicotinamide adenine dinucleotide (NAD^+^)‐dependent deacetylases (Wan et al., 2023). SIRT1 interferes with various biological events, such as energy metabolism, apoptosis, ER stress, and increases the cell's antistress ability that results in cell survival (Divya & Ravanan, 2023). ER stress and SIRT1 interaction has been involved in multiple pathological processes including metabolic, inflammatory, neurodegenerative, and neoplastic diseases (Wang et al., 2020). Activation of SIRT1/Nrf2 pathway was suggested to attenuate oxidative injury and ER stress‐induced apoptosis through increasing Bcl‐2 expression while decreasing Bcl‐2‐like protein (Bax), caspase 3, GRP78, and CHOP expression (Wang et al., 2019; Yarmohammadi et al., 2021). miR‐34a is negatively influenced by SIRT1 function (Fu et al., 2017). Evidence showed that miR‐34a is upregulated during myocardial I/R injury through the Kelch‐like ECH‐associated protein 1 (Keap1)/Nrf2 signaling pathway that consequently elevates the antioxidant enzymes (Yarmohammadi et al., 2023). In this regard, Wang et al. (2019) showed that crocin (10 μM) decreased miR‐34a expression, while it increased SIRT1, Nrf2, and heme oxygenase‐1 (HO‐1) levels in I/R‐induced injury in cardiomyocytes. Hence, crocin was suggested to reduce ER stress and apoptotic cell death as well as improve cardiac function by modulating the miR‐34a/SIRT1/Nrf2 signaling pathway (Wang et al., 2019). Endothelial and vascular smooth muscle cells play a critical role in atherosclerotic plaque formation that causes the LDLs' migration into the intimal layer and their subsequent oxidation to oxidized LDL (Ox‐LDL) (Kattoor et al., 2019). Ox‐LDLs then trigger macrophage recruitment and formation of foam cells, which would enhance the initiation and progression of atherosclerosis (Kattoor et al., 2019; Khatana et al., 2020). He et al. (2005) investigated the effect of crocin on atherosclerosis in an experimental model of atherosclerosis in the quails fed with hyperlipidemic diet. They showed that crocin administration can reduce the level of serum TG, TC, and low‐density lipoprotein‐cholesterol (LDL‐C) and inhibit the aortic plaque formation, which are involved in the progression of atherosclerosis. In addition, crocin lowered MDA and serum level of NO in the quails. In the bovine aortic endothelial cells incubated with Ox‐LDL (50 mg/L), treatment with crocin exhibited an inhibitory effect on the activities of lactate dehydrogenase (LDH), nitric oxide synthase (NOS), and NO. Crocin also inhibited proliferation of the cultured bovine aortic smooth muscle cells exposed to 100 μg/L of Ox‐LDL. Hence, crocin is supposed to modulate cell apoptosis and redox hemostasis (He et al., 2005). Activation of the smooth muscle cells, characterized by a hyperpolarized state, depends on Ca^2+^ concentration. Based on evidence from confocal microscopy, crocin (10^−8^, 1 × 10^−7^, 1 × 10^−6^ mol/L) concentration‐dependently decreased the intracellular Ca^2+^ concentration in hydrogen peroxide (H_2_O_2_, 1 × 10^−2^ mol/L)‐treated cells (He et al., 2004). This effect of crocin may be related to inhibition of the extracellular Ca^2+^ influx and release of intracellular Ca^2+^ stores. Therefore, antioxidant activity and normalizing function of endothelial and smooth muscle cells contribute to anti‐atherosclerotic effect of crocin. Another study also proposed the protective effects of crocin against high glucose (33 mmol/L)‐induced injury in human umbilical vein endothelial cells (HUVECs) (Zhang et al., 2018). As previously shown, high extracellular glucose can activate ER stress response that consequently causes vascular injury, as characterized by an increase in the apoptotic cell number, inflammatory cytokine, intracellular ROS level, as well as a decrease in the expression of vascular endothelial growth factor A (VEGF‐A) (Madonna et al., 2018; Ricciardi & Gnudi, 2020). Zhang et al. (2018) showed that pretreatment of HUVECs with crocin or 4‐phenylbutyrate (ER stress inhibitor) exerted antioxidative, antiapoptotic, anti‐inflammatory, and pro‐angiogenic effects that would rely on the modification of ER stress (Zhang et al., 2018).

## 5 APOCAROTENOIDS TARGET ER IN CANCERS

Cancer is the second biggest cause of mortality worldwide, after cardiovascular disease (Mahase, 2019). Hence, a large number of researches focused on the discovery and development of new drugs that increase patients' survival with minimal side effects (Anwanwan et al., 2020). Several in vitro, in vivo, and clinical studies have showed the anticancer effects of apocarotenoids (Heidarzadeh et al., 2018; Leone et al., 2018). The saffron carotenoids were found to upregulate pro‐apoptotic factors such as p53 and Bax, as well as induce cell cycle arrest in G1 or G2 phases, thereby reducing cell survival and tumor growth (Veisi et al., 2020). Furthermore, the antitumor activities of these phytochemicals may improve the toxicity effects of chemotherapy agents and radiation on cancer cells (Leone et al., 2018). Apocarotenoids were shown to induce apoptosis in some cancerous cells (Leone et al., 2018). For instance, treatment of breast cancer MDA‐MB‐468 cell line with crocin could upregulate caspase‐9 expression as well as induce the splicing of XBP1 mRNA and microtubule‐associated protein 1A/1B‐light chain 3 (LC3)‐phosphatidylethanolamine conjugate (LC3‐II) accumulation. Accordingly, activating ER‐related autophagy and apoptotic pathways were involved in the cytotoxic and anticancer effects of crocin (Heidarzadeh et al., 2018). In BT‐474, a known HER2+ breast cancer cell line, crocin (3.5 mg/mL) induced apoptosis, as indicated by an increase in cleaved caspase‐9 expression. Considering XBP1 gene splicing following crocin treatment, the activation of UPR was suggested to have an important role in the anticancer effects of crocin (Nassim et al., 2019). In another study, safranal suppressed DNA repair with subsequent DNA damage as well as inhibited histone‐H3 phosphorylation, Cyclin B1 and cell dvision cycle 2 (Cdc2), reflecting cell cycle arrest in the HepG2 cells. Furthermore, the antiproliferative effects of safranal were associated with upregulation of GRP78, PERK, IRE1, and ATF6, suggesting the involvement of ER‐mediated cell death (Al‐Hrout et al., 2018). The authors proposed that persistent ER stress by safranal may activate caspase‐8 and ‐9 (initiator caspases) with subsequent cleavage of caspase‐3 and ‐7 (executioner caspases), poly (adenosine diphosphate (ADP)‐ribose) polymerase (PARP), and ultimately apoptosis (Al‐Hrout et al., 2018).

## 6 APOCAROTENOIDS COUNTERACT ER STRESS IN RESPIRATORY SYSTEM DISORDERS

Asthma is regarded as the most common complex chronic inflammatory disorder of the lung (Lambrecht & Hammad, 2015). The inflammatory response is driven by the recruitment of various immune cell types that consequently lead to lung injury, goblet cell hyperplasia, and fibrosis (Habib et al., 2022). Recently, saffron supplementation was found to effectively improve clinical symptoms of patients with allergic asthma (Zilaee et al., 2019). Some scholars investigated the possible mechanisms behind the protective effects against allergic asthma using in vitro and in vivo experiments (Aslani et al., 2022; Boskabady & Aslani, 2006; Kianmehr & Khazdair, 2020). For instance, the study by Aslani et al. (2022) demonstrated that administration of crocin (25, 50, and 100 mg/kg) to ovalbumin (OVA)‐sensitized mice ameliorated airway inflammation, which was comparable to that of dexamethasone. Crocin also suppressed caspase‐12 and CHOP expression in lung tissue (Aslani et al., 2022). Accordingly, crocin was supposed to attenuate ER stress and subsequent apoptosis in the lung tissue. In another study, an aqueous‐ethanolic extract *C. sativus* (saffron) and safranal showed a relaxant effect on guinea‐pig precontracted tracheal chain, which was comparable to that of theophylline. The possible mechanism of the relaxant effect was supposed to take place through inhibition of Ca^2+^ channel or sarcoplasmic reticulum Ca^2+^ release into the cytosol. However, the exact mechanism behind such action needs to be investigated (Boskabady & Aslani, 2006).

## 7 APOCAROTENOIDS COUNTERACT ER STRESS IN OCULAR DISORDERS

Until now, the influence of apocarotenoids on ER function in ocular diseases has been the subject of one study (Yamauchi et al., 2011). Apocarotenoids were found to exert promising antioxidant effects on the oxidative stress‐mediated ocular disorder. Retinal damage involves retinal ganglion cell death, photoreceptor degeneration, and retinal dysfunction (Almasieh et al., 2012; Soldatov et al., 2021). Administration of crocetin (100 mg/kg, oral) significantly attenuated light‐exposure‐induced retinal damage in vivo. Furthermore, Yamauchi et al. (2011) revealed that crocetin (3 μM) suppressed the expression of BiP and CHOP, caspase‐3 and ‐9 in the retinal ganglion cells treated with and tunicamycin‐ and H_2_O_2_, as an inducer of oxidative stress. These results suggest that crocetin alleviates ER disturbance and subsequent apoptotic cell death in retinal cells (Yamauchi et al., 2011).

## 8 APOCAROTENOIDS COUNTERACT ER STRESS IN OSEOARTRITIS

Osteoarthritis (OA), the most common disease of joints in adults, is regarded as an age‐related degenerative disease (Musumeci et al., 2015). Oxidative stress is involved in the pathogenesis of osteoarthritis, which mediates chondrocyte apoptosis and extracellular matrix (ECM) degeneration through the activation of ER stress (Zahan et al., 2020). Apocarotenoids possess antioxidant capacity and may thus be helpful in the treatment of osteoarthritis (Zahan et al., 2020). Safranal administration (intraperitoneal injection of 90 and 180 mg/kg) prevented the development and progression of OA in mice. In the cultured osteocytes, safranal (30 μM) *tert*‐butyl hydroperoxide counteracted ROS overproduction and oxidative stress. The inhibitory effects of safranal on chondrocyte apoptosis and extracellular matrix degeneration were associated with upregulated Sirt1 expression while decreasing PERK–eIF2α–CHOP. Hence, safranal exerts its beneficial effects through modulating ER stress‐related sensors in the cells (Zhang et al., 2022).

## 9 POTENTIAL PROTECTIVE EFFECTS OF APOCAROTENOIDS AGAINST XENOBIOTICS TOXICITY AND ISCHEMIC INJURY

A large number of studies showed that apocarotenoids have considerable protective effects against a wide range of xenobiotics, such as drugs, toxins, chemical agents, and heavy metals (Rajabian et al., 2019; Zarei & Elyasi, 2022). During the last decade, evidence showed the role of ER stress in toxic elements‐induced toxicity (Rana, 2020). The mechanisms underlying these protective activities of saffron carotenoids against toxic elements involve the inhibition of oxidative stress, inflammation, and apoptosis, and cell structure stabilization (Ghaffari & Roshanravan, 2019; Leone et al., 2018; Zarei & Elyasi, 2022). Apart from mitochondria, regulation of ER function was identified as one of the major targets of saffron carotenoids in the stress conditions. Considering liver is a common site of exposure to many chemicals (Rana, 2020), many investigations focused on the hepatoprotective mechanisms of apocarotenoids. A previous study showed that administration of saffron extract (60 mg/kg) alleviated hepatotoxicity induced by copper nanoparticles (100 and 250 mg/kg) in mice through attenuating oxidative changes of the key organelles in cytoplasm such as mitochondria and ER (Attia et al., 2021). D‐Galactose is a reducing sugar that, at high levels and chronic systemic administration, leads to oxidative stress, inflammation, apoptosis, and mimicking the natural aging process in rodents (Azman & Zakaria, 2019; Mirzavi et al., 2022). Lipopolysaccharide (LPS) is an endotoxin from gram‐negative bacteria that activates the innate immune response during sepsis (Bardaghi et al., 2023; Pfalzgraff & Weindl, 2019). The uncontrolled hyperinflammatory responses and oxidative stress trigger apoptotic cell death that is associated with multiple organ failure following LPS‐induced sepsis (Hassan et al., 2020). The results of a previous study exhibited that administration of crocetin to LPS–D‐galactose‐injured rats successfully regulated the expression of apoptosis‐related molecules inducing caspase‐12, 9, 8, and 3, Bax, Bcl‐2 interacting mediator of cell death (Bim), BH3‐interacting domain death agonist (Bid), and p53 in liver tissue. Caspase‐12 is specifically activated following ER stress (de la Cadena & Massieu, 2016). Hence, the protective effects of crocetin against ER stress play an important role in attenuating oxidative stress injury induced by LPS/D‐galactosamine (Gao et al., 2019; Pashirzad et al., 2019). In another study, saffron ethanolic extract protected liver against I/R injury. The extract restored antioxidant status as reflected by reduction in ROS production and protein oxidation while elevating SOD level. Meanwhile, it suppressed cleaved caspase‐3 and GRP78, which were dysregulated during I/R. Therefore, attenuation of oxidative stress might help to attenuate ER stress and apoptotic cell death in hepatic I/R injury (Malhi & Kaufman, 2011; Pan et al., 2013). Ben Salem et al. (2015) showed the protective effects of crocin against Zearalenone‐induced toxicity and ER stress in human colon carcinoma (HCT116) and embryonic kidney (HEK293) cells. α‐ and β‐Zearalenol are the major metabolites of Zearalenone, a nonsteroidal estrogenic mycotoxin (Wu et al., 2021), inducing ER stress as evidenced by the upregulated GRP78 and GADD34 proteins (Feng et al., 2022; Wu et al., 2021). Activation of the ER stress response is associated with the mitochondrial‐dependent apoptosis, ROS overproduction, and oxidative stress. Pretreatment of HEK293 cells with crocin (250 μM) before zearalenol exposure downregulated GRP78 and GADD34, CHOP while upregulating ATF4 and protein disulfide isomerase A6 (PDIA6). Accordingly, crocin attenuated the ER stress and subsequent apoptosis (Ben Salem et al., 2015). Crocin was also shown to attenuate toxicity induced by patulin, a mycotoxin and natural contaminant of fruits produced by *Penicillium*, *Aspergillus*, and *Byssochlamys* (Rossi et al., 2021; Vidal et al., 2019). Based on toxicological evidence, patulin exerts gastrohepatotoxicity, nephrotoxic, neurotoxic, and immunotoxic effects (Ramalingam et al., 2019). The results of a previous study also showed that patulin upregulated GRP78 and GADD34 expressions in the HCT116 and HEK293 cells. The antioxidant effects of crocin (250 μM) were proposed to attenuate lipid peroxidation and subsequent ER stress in this study (Boussabbeh et al., 2016). As mentioned earlier, SIRT1 is regarded as a sensor of cellular energy status and oxidative stress in various tissues, which is interlinked with ER stress (Wang et al., 2020). In an in vitro model of the pubertal testis, pre‐ and posttreatment with crocetin (50 μM) restored SIRT1, p62, and LC3‐II expression, which was associated with improvement in oxidative DNA damage and antioxidant levels in irradiation‐induced injury (Li, Feng, et al., 2020). A detailed summary of the protective effects of apocarotenoids in ER‐stress‐related diseases is presented in Table 1.

**TABLE 1:** A detailed summary of the protective effects of apocarotenoids in ER‐stress‐related diseases.

Disorder	Experimental model	Results	Reference
Multiple sclerosis	In vivo: autoimmune encephalomyelitis model using C57BL/6 wild and CHOP2/2 mice, Crocin (100 mg/kg) In vitro: human fetal astrocytes (200 mM)	Suppression of XBP‐1, BiP, PERK, and CHOP, and TNF‐α in the spinal cords Suppression of Syncytin‐1	Deslauriers et al. (2011)
Alzheimer's disease	In vivo: a rat model induced by Aβ_25–35_, crocin (40 mg/kg)	Amelioration of learning and memory acquisition using Y‐maze test Inhibition of ER stress and apoptosis of brain tissue Reduction in GRP78 and CHOP	Lin et al. (2019)
Ischemia/reperfusion	In vitro: cerebral ischemia/reperfusion‐like condition in oxygen glucose‐deprived SH‐SY5Y cells, crocetin In vivo: a rat model of vascular dementia, crocetin	Attenuating ER stress and neuronal apoptosis Suppression of the PERK/eIF2α/ATF4/CHOP pathway	Yuan et al. (2023)
Traumatic brain injury	In vivo: rat, crocetin (500 mg/kg)	Attenuation of neuronal apoptosis Upregulation of Bcl2 Suppression of several factors promoting the release of calcium (Ca^2+^) from ER	Bie et al. (2011)
Parkinson's disease	In vitro: MPP^+^‐induced neuron‐like PC12 cells' injury, crocin (0.1, 1, 10, or 100 μM)	Regulating CHOP/Wnt pathway ER‐related apoptosis	Zhang et al. (2015)
Neurotoxicity	In vivo: Sofosbuvir‐induced neurotoxicity, saffron extract treatment (80 mg/kg body weight/day, 6 weeks)	Attenuation of neurotoxicity‐induced oxidative stress Amelioration of ER alteration	Ibrahim et al. (2021)
	In vivo: copper oxide nanoparticles' neurotoxicity, crocin (30 mg/kg/day for 14 days)	Attenuation of neurotoxicity‐induced oxidative stress through suppression of lipid peroxidation and upregulation of antioxidant defense system including SOD, catalase, GPX, and glutathione	Mohamed Mowafy et al. (2021)
Diabetes	In vivo: STZ‐injected rats, aqueous extract of saffron	Hypoglycemic and hepatoprotective effects Normalization of blood glucose and serum and liver total lipids and oxidative status (malondialdehyde and hydrogen peroxide level) Amelioration of ER and mitochondrial alteration	Ali et al. (2016)
	In vivo: glyoxalase 1‐ in methylglyoxal‐induced diabetic mice, crocin (15, 30, and 60 mg/kg)	Ameliorating fasting blood glucose and redox hemostasis (MDA, glutathione, and glyoxalase 1–Nrf2). Suppression of miRNAs such as miR‐204, miR‐216b, miR‐192, and miR‐29a expression and subsequent ER stress	Radmehr et al. (2022).
Cardiovascular disease	In vivo: mouse model of I/R‐induced injury In vitro: I/R‐induced primary cardiomyocytes, crocin (10 μM)	Upregulation of Sirtuin1 and Nrf2, HO‐1 and downregulation of miR‐34a expression Attenuation of ER stress‐induced apoptosis through increasing Bcl‐2 and decreasing Bax, caspase 3, GRP78, and CHOP expression	Wang et al. (2019).
	In vivo: quails fed with hyperlipidemic diet, crocin (25, 50, and 100 mg/kg) In vitro: cultured bovine aortic smooth muscle and endothelial cells incubated with Ox‐LDL (50 mg/L), crocin (10^−8^, 1 × 10^−7^, 1 × 10^−6^ mol/L)	Reduction in serum TC, TG, LDL‐C, MDA and formation of aortic plaque Inhibition of proliferation of the cultured cells	He et al. (2005)
	In vitro: H_2_O_2_ (1 × 10^−2^ mol/L)‐treated cells, crocin (10^−8^, 1 × 10^−7^, 1 × 10^−6^ mol/L)	Inhibition of the extracellular Ca^2+^ influx and release of intracellular Ca^2+^ stores	He et al. (2004)
	In vitro: HUVECs exposed to high extracellular glucose, crocin	Antioxidative, antiapoptotic, anti‐inflammatory, and proangiogenic effects similar to 4‐phenylbutyrate (ER stress inhibitor)	Zhang et al. (2018)
Respiratory system dysfunction	In vivo: ovalbumin‐sensitized mice, crocin (25, 50, and 100 mg/kg)	Amelioration of airway inflammation through suppression of ER stress and apoptosis (downregulation of caspase‐12 and CHOP) in lung	Aslani et al. (2022)
	In vivo: an aqueous‐ethanolic extract (0.15, 0.3, 0.45, and 0.60 g %) and safranal (0.15, 0.30, 0.45, and 0.60 mL–0.2 mg/mL solution)	A relaxant effect on guinea‐pig precontracted tracheal chain through inhibition of Ca^2+^ channel or sarcoplasmic reticulum Ca^2+^ release into the cytosol	Boskabady and Aslani (2006)
Retinal damage	In vitro: crocetin (3 μM) in the retinal ganglion cells treated with and tunicamycin‐ and H_2_O_2_ In vivo: crocetin (100 mg/kg in light‐exposure‐induced retinal damage)	Attenuation of ER disturbance and apoptosis through downregulation of BiP and CHOP, caspase‐3 and ‐9	Yamauchi et al. (2011)
Osteoarthritis	In vivo: Safranal (90 and 180 mg/kg) in mice. In vitro: osteocytes, safranal (30 μM)	Inhibition of ROS and oxidative stress in chondrocyte Inhibition of apoptosis and extracellular matrix degeneration through upregulating Sirt1 and decreasing PERK–eIF2α–CHOP expression	Zhang et al. (2022)
Cytotoxic and anticancer effects	In vivo: hepatotoxicity induced by copper nanoparticles (100 and 250 mg/kg) in mice, saffron extract (60 mg/kg)	Attenuating oxidative changes of mitochondria and ER	Attia et al. (2021)
	In vitro: MDA‐MB‐468 cell, crocin (1–5 mg/mL)	Activation of ER‐related autophagy and apoptotic pathways through upregulating caspase 9 and splicing of XBP1 mRNA and LC3‐II accumulation	Heidarzadeh et al. (2018)
	In vitro: BT‐474 cell, crocin (3.5 mg/mL)	Activation of UPR (XBP1 gene splicing) and apoptosis (upregulation of cleaved caspase‐9)	Nassim et al. (2019)
	In vitro: HepG2 cells, safranal (30–100 μM)	Antiproliferative and apoptotic effects through DNA damage, cycle arrest (Cyclin B1 and Cdc2), activating caspase cascade (caspase‐8 and ‐9, ‐3, and ‐7, PARP) ER stress through upregulation of GRP78, PERK, IRE1, and ATF6	Al‐Hrout et al. (2018)
	In vivo: LPS–D‐galactose‐injured rats, crocetin stress injury induced by LPS/D‐galactosamine	Inhibition of ER stress and apoptosis through modulating caspase‐12, 9, 8, 3, Bax, Bim, Bid, and p53 in liver	Gao et al. (2019)
	In vivo: I/R‐induced injury in liver, saffron ethanolic extract	Redox hemostasis by reduction in ROS and protein oxidation and elevating SOD ER stress and apoptotic cell death through regulating caspase‐3 and GRP78	Malhi and Kaufman (2011)
	In vitro: Zearalenone‐induced toxicity in HCT116 and HEK293 cells, crocin (250 μM)	Attenuating ER stress through downregulation of GRP78 and GADD34, and CHOP, along with upregulation of ATF4 and PDIA6 Inhibition of mitochondrial‐dependent apoptosis, ROS overproduction, and oxidative stress	Ben Salem et al. (2015)
	In vitro: Patulin‐induced toxicity, in the HCT116 and HEK293 cells, crocin (250 μM)	Inhibition of oxidative stress and ER stress through upregulation of GRP78 and GADD34 expressions	Boussabbeh et al. (2016)
	In vitro: irradiation‐induced injury in pubertal testis, crocetin (50 μM)	Restoring SIRT1, p62, and LC3‐II expression and redox hemostasis	Li, Feng, et al. (2020)

**FIGURE 3:**
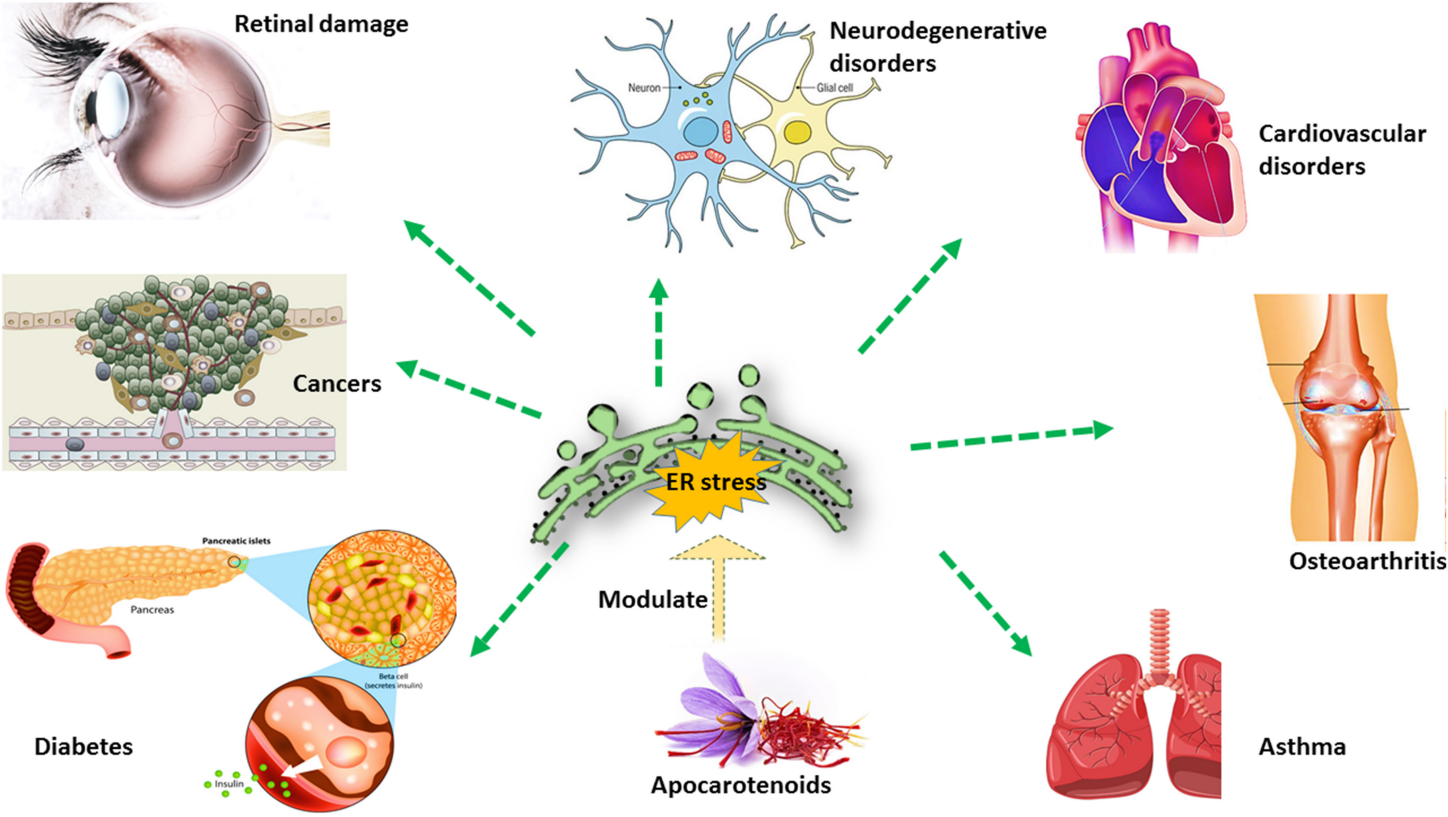
Protective effects of apocarotenoids on various ER stress‐related disorders.

As discussed earlier, saffron apocarotenoids have different therapeutic functions in various diseases, including neurological diseases, cardiovascular diseases, osteoarthritis, and metabolic disorders. These pathological conditions can be developed due to inflammation, and activation of cell death pathways, and impaired biochemical homeostasis (Roshanravan & Ghaffari, 2022). Interestingly, ER stress can establish a progressive pathological cycle with oxidative stress and inflammation (Li, Cao, et al., 2020). Therefore, antioxidants such as saffron can play an important role in preventing and limiting these disorders. The evidences demonstrated that saffron apocarotenoids might exert protection in the diseases by inhibiting inflammation, excitotoxic pathway, autophagy regulation, inhibition of apoptosis, and modulation of oxidative status (Roshanravan & Ghaffari, 2022). Figure 3 illustrates the therapeutic potential of apocarotenoids against various ER stress‐related disorders. The results from clinical studies proposed the potential of apocarotenoids for reversing the progressive pathological process (El Midaoui et al., 2022; Ghiasian et al., 2019; Nanda & Madan, 2021). Ghiasian et al., 2019 examined the potential protective effects of crocin in the multiple sclerosis patients and demonstrated a remarkable reduction in the levels of TNF‐α, interleukin 17 (IL‐17), lipid peroxidation, and DNA damage (Ghiasian et al., 2019). Interestingly, an increase was observed in the autopsied brain specimens from MS individual, XBP‐1 spliced variant (XBP‐1/s) confirming the involvement of ER stress in the MS pathology. Previous evidence support the idea that neuro‐inflammation and ER stress contribute to the pathogenesis of demyelination and neurodegeneration (Deslauriers et al., 2011). In this study, Syncytin‐1 was found to induce a robust effect on XBP‐1 splicing in human astrocytes, which was partially inhibited by crocin (Deslauriers et al., 2011). Studies investigating the effectiveness of saffron and apocarotenoids on the progression of neurodegenerative diseases showed that they could be an appropriate option to slow the progression of neurodegenerative diseases due to its strong antioxidant and anti‐inflammatory effects (Ghasemi Sakha et al., 2020; Ghiasian et al., 2019; Gudarzi et al., 2022). However, limited information is available to make any suggestion regarding the effect of apocarotenoids on ER stress‐related pathological pathways. Hence, human studies targeting ER stress are needed to evaluate the protective effects of saffron apocarotenoids.

## 10 CONCLUSIONS

Based on in vitro and in vivo evidences (as summarized in Table 1), apocarotenoids have potential protective effects against various ER‐related pathological conditions, such as cardiovascular, metabolic, inflammatory, ischemia, and organ damage induced by toxic compounds (e.g., aluminum, MPP^+^, D‐galactose, and LPS). ROS and other toxic compounds lead to accumulated/misfolded proteins and subsequent ER stress and persistent ER stress activate apoptotic cell death. Apocarotenoids ameliorate oxidative stress via decreasing ROS, modulating Nrf2/HO‐1, and restoring glutathione and SOD levels. Moreover, they exert its ER protecting properties by modulating GADD34, GRP78, PERK, eukaryotic initiation factor‐2α (e‐IF2α), AFT4/6, and XBP‐1. Recently, SIRT1 and miRNAs have emerged as novel targets of apocarotenoids. They modulate expression of miR‐204, miR‐216, miR‐192, miR‐29, and miR‐34a in pancreas and heart tissue. Apocarotenoids are associated with biological properties on ER stress and related markers and can be a potential therapeutic candidate for various disorders.

## AUTHOR CONTRIBUTIONS

**Farshad Mirzavi:** Conceptualization (equal); writing – original draft (equal). **Arezoo Rajabian:** Conceptualization (equal); writing – original draft (equal). **Hossein Hosseini:** Writing – review and editing (lead).

## ACKNOWLEDGEMENTS

This research received no external funding.

## CONFLICT OF INTEREST STATEMENT

The authors declare no conflicts of interest.

## Data Availability

Data sharing is not applicable to this article as no new data were created or analyzed in this study.
